# Mesenchymal stem cells-derived extracellular vesicles as a novel drug delivery carrier: engineering strategies and clinical safety estimation

**DOI:** 10.3389/fmolb.2026.1838554

**Published:** 2026-06-18

**Authors:** Na Liu, Xue Zhao, Xicai Sun, Yongmei Liu, Jing Xu, Donghua Xu, Wenchang Sun

**Affiliations:** 1 Center of Medical Research, Weifang People’s Hospital, Shandong Second Medical University, Weifang, Shandong, China; 2 Department of Clinical Laboratory, Weifang People’s Hospital, Shandong Second Medical University, Weifang, Shandong, China; 3 Department of Hospital Office, Weifang People’s Hospital, Shandong Second Medical University, Weifang, Shandong, China; 4 Department of Gynecology, Weifang People’s Hospital, Shandong Second Medical University, Weifang, Shandong, China; 5 Department of Rheumatology and Immunology, Weifang People’s Hospital, Shandong Second Medical University, Weifang, Shandong, China

**Keywords:** drug carriers, engineering, extracellular vesicles, mesenchymal stem cells, preconditioning

## Abstract

Extracellular vesicles derived from mesenchymal stem cells (MSC-EVs) have biocompatibility and low immunogenicity. They can effectively mediate intercellular communication by transmitting various information molecules, including polypeptides, lipids, and nucleic acids, thereby demonstrating unique potential in the field of drug delivery. Given their crucial roles in immune regulation and regenerative medicine, an increasing number of studies are focusing on developing MSC-EVs as drug carriers. This paper presents a systematic review of the future development directions of MSC-EVs as drug carriers, with a particular emphasis on their origins and engineering modification or pretreatment strategies. By employing engineering techniques to modify MSC-EVs, it is possible to precisely regulate key properties such as drug-loading capacity and targeting ability, thereby providing a novel approach for efficient drug delivery. Meanwhile, this paper delves into the safety evaluation of MSC-EVs in clinical applications across different diseases, comprehensively assessing their safety and efficacy as drug carriers based on clinical studies in the fields of lungs, skin, nervous system, eyes, and gastrointestinal tract, among others. Furthermore, we provide an outlook on the potential value and challenges associated with the clinical application of MSC-EVs as drug carriers in various diseases in the future, aiming to offer theoretical support and research directions for promoting MSC-EVs as safe and effective drug carriers.

## 1 Introduction

Mesenchymal stem cells (MSCs) are multipotent stem cells characterized by strong proliferative capacity and the potentials to differentiate into various cell types (Russell et al., 2010). MSCs are derived from a diverse array of tissues, including adipose tissue, peripheral blood, bone marrow, dental pulp, placental tissue, umbilical cord tissue, and cord blood (Hass et al., 2011). Under suitable conditions, MSCs possess the capacity to differentiate into various cell types, such as osteoblasts, chondrocytes, adipocytes, neurons, hepatocytes, and endothelial cells. The immunomodulatory and regenerative capabilities of MSCs render them effective in the treatment of many kinds of diseases, particularly the refractory diseases. More than a dozen MSCs-based therapies have presently been approved for commercial use globally (Fernández-Garza et al., 2023). MSC-EVs are enclosed by a phospholipid bilayer, which are generated through either budding or the fusion of multivesicular bodies with the plasma membrane. These vesicles can be classified based on the size into exosomes (40–160 nm), microvesicles (200–800 nm), and apoptosis bodies (1–5 μm) (Thakur et al., 2022a; Thakur et al., 2022b). The dimensions of MSC-EVs alter with the formation mechanisms and cellular origins. Notably, apoptosis bodies can be internalized by MSCs, thereby modulating signaling pathways that are crucial for stem cell regulation. Microvesicles and exosomes are internalized via multiple mechanisms, including lipid raft-, caveolar-, and clathrin-mediated endocytosis, as well as receptor-mediated signal transduction (Liu et al., 2018).

MSC-EVs are recognized as vectors for the transmission of biological information between MSCs and target cells by delivering bioactive molecules, such as peptides, lipids, and nucleic acids, thereby regulating the growth, differentiation, and apoptosis and other biological processes of target cells (Kesidou et al., 2024; Chen et al., 2019). Accumulating evidence indicates that MSCs exert paracrine effects via MSC-EVs, participating in immune modulation, tissue regeneration, repair, and anti-tumor responses (Zhou et al., 2024). In particular, MSC exosomes have been documented to confer effects through complicated signaling pathways, including nuclear factor kappa-B (NF-κB), nucleotide-binding oligomerization domain 2 (NOD2), and yes-associated protein 1 (YAP1) (Chen et al., 2023; Wang Y. et al., 2024; Banerjee et al., 2024). Furthermore, MSC exosomes possess the ability to traverse the blood-brain barrier and are more readily internalized by recipient cells owing to their homing capabilities, thereby enhancing their biological efficacy in specific organs and tissues (Thakur et al., 2020a; Thakur et al., 2020b). In terms of immune regulation, prostaglandin E2 encapsulated by MSC-EVs has been shown to inhibit the maturation and inflammatory responses of dendritic cells (Zagoura et al., 2012; Harrel et al., 2019). Exosomes derived from umbilical cord MSCs (UC-MSC exosomes) are enriched with hsa-mir-21 and hsa-mir-328-3p, which facilitate the osteoblast differentiation (Yahao and Xinjia, 2021; Jiang et al., 2021).

It is worth mentioning that MSC-EVs demonstrate immense application potential in the field of drug delivery and can serve as an efficient drug carrier. It is capable of carrying drug molecules to smoothly penetrate various biological barriers, enabling precise and targeted drug delivery as well as controlled release within the body. This characteristic not only significantly enhances drug efficacy, but also effectively reduces drug side effects, bringing about new breakthroughs in drug therapy. For instance, miR-223 delivering by MSC exosomes can promote wound healing via inducing M2-type macrophage polarization, thereby inhibiting macrophage-mediated inflammation (He et al., 2019). Exosomes derived from induced pluripotent stem cells (iPSC exosomes) significantly enhance endothelial cell proliferation and migration by activating the PI3K/Akt signaling pathway, thereby improving angiogenesis (Liu et al., 2017). MiR-138-5p within MSC exosomes has been shown to inhibit the proliferation and migration of skin fibroblasts through the downregulation of silent information regulator 1 (SIRT1), thereby reducing pathological scar formation and promoting extracellular matrix remodeling during skin wound repair (Zhao et al., 2022; Wang et al., 2017). UC-MSC exosomes facilitate skin wound healing and angiogenesis by transferring angiopoietin 2 (Liu et al., 2021). Additionally, gingival MSC exosomes enhance re-epithelialization, collagen deposition and remodeling when incorporated into hydrogel sponges, which also promote the angiogenesis and neuronal ingrowth, thereby contributing to the healing of skin wounds in diabetic rat models (Sun et al., 2022; Tian et al., 2022; Shi et al., 2017). MSC-EVs therapy presents no significant risk of immune rejection, thereby offering a safer and more efficacious treatment option with extensive applications in regenerative medicine and immunotherapy (Golchin et al., 2019; Hai et al., 2022). As the principal paracrine mechanism of MSCs, MSC-EVs possess substantial biological effects with several advantages, including high biocompatibility, low immunogenicity, robust bio-targeting capabilities, and easy to storage and transmission.

Given the immense potential of MSC-EVs, this paper focuses on the future development directions of MSC-EVs as drug carriers and provides an in-depth review of the latest progress in clinical research on their application in various diseases. On the one hand, it elaborates on several innovative strategies that effectively enhance the therapeutic effects of functionalized MSC-EVs through engineering modifications or pretreatment methods of MSCs. On the other hand, it comprehensively analyzes the latest MSC-EVs-related clinical studies, conducting a thorough and systematic evaluation of their safety and efficacy in clinical research. The aim is to offer robust theoretical support and practical guidance for promoting the widespread clinical application of MSC-EVs as drug carriers.

## 2 The biological occurrence and origins of MSC-EVs

MSC exosomes are mainly produced through the endocytic pathway, involving the formation and release of multivesicular bodies (MVBs). After the endoplasmic reticulum and Golgi apparatus of MSCs synthesize proteins and lipids, these components are encapsulated into endosomes to form MVBs. Subsequently, MVBs fuse with the cell membrane and release exosomes into the extracellular space (Yin K. et al., 2019). Microvesicles are formed by budding directly from the cell membrane and then detaching (Carvalho Ferraz et al., 2025). Apoptotic bodies are formed during the process of cell apoptosis. During this process, the cell membrane undergoes asymmetrical changes and breaks down to form larger vesicles, which contain cellular organelle fragments or nuclear components (Yufan Zhu et al., 2023). MSC-EVs can be distinguished from EVs derived from other cell types by their distinct surface protein profiles. In addition to tetraspanins (CD9, CD63, CD81) that are common to most EVs, MSC-EVs carry MSC-characteristic surface markers including CD44, CD73, CD90, and CD105, while lacking hematopoietic cell markers such as CD34 and CD45 (Witwer et al., 2019). Recent studies have proposed CD44, CD73, and CD105 as positive markers for the minimal identification of MSC-EVs (Nguyen et al., 2024). Furthermore, MSC-EVs carry high levels of immunomodulatory molecules, such as TGF-β, HGF, PD-L1, and galectin-1, and are rich in miRNAs (such as miR-21, miR-146a, miR-181c) that regulate inflammatory, apoptotic and metabolic pathways (Qiu et al., 2019). MSCs exhibit obvious heterogeneity in their proliferation, apoptosis, and differentiation, resulting in differences in the biological effects (Zhang Y. et al., 2018; Lee et al., 2019). MSCs can be isolated from diverse human tissues, with bone marrow, adipose tissue, and umbilical cord being the most prevalent sources. The selection of MSCs is critical and should take into account some key factors, such as the collection site, timing, harvesting technique, and potential postoperative complications. It has been well demonstrated bone marrow-derived MSCs (BM-MSCs) are more widely used for the treatment of brain and spinal cord injuries due to their elevated expression of neurotrophic factors, such as brain-derived neurotrophic factor (BDNF) and nerve growth factor (NGF), as well as the potency of neural lineage differentiation. The adipose tissue-derived MSCs (AT-MSCs) are more suitable for reproductive disorders and skin regeneration, given the generation of adipose-derived growth factors and cytokines that promote dermal and epidermal regeneration. UC-MSCs are more suitable for lung diseases and acute respiratory distress syndrome (ARDS) due to their immunomodulatory capacity, low immunogenicity and efficacy in suppressing pulmonary inflammation and fibrosis (Hoang et al., 2022). Moreover, UC-MSCs are extensively utilized in clinical trials owing to their relatively easy procurement through non-invasive methods, high differentiation potentials, and robust regenerative capabilities, rendering them promising candidates for regenerative medicine (Jo et al., 2021; Naji et al., 2019).

## 3 MSC-EVs acquirement and engineered processing

**FIGURE 1:**
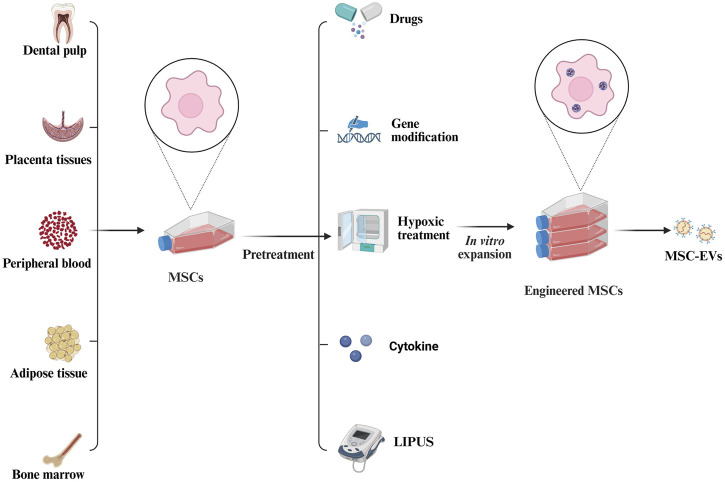
The engineering process of MSCs. (The figure was created with Biorender.com).

EVs represent promising natural vectors for drug delivery. Their roles in drug delivery have been extensively investigated during the last decade. As nanoscale vesicles, they possess the capability to traverse diverse biological barriers, thereby exerting significant biological effects in local microenvironment. The rationale for employing engineered over native MSC-EVs is multifold. First, native MSC-EVs exhibit limited tissue-specific targeting ability, relying primarily on passive biodistribution and non-specific cellular uptake. In contrast, the engineered MSC-EVs can more effectively target specific tissues or cell types, significantly enhancing local drug concentrations at the disease site (Meng et al., 2023). Second, the cargo-loading capacity of native MSC-EVs is inherently limited and dependent on the physiological state of the parental MSCs. Through preconditioning or genetic modification, the payload of therapeutic molecules-including specific miRNAs, proteins, and chemical drugs-can be substantially enriched in MSC-EVs, amplifying their therapeutic potency (Phan et al., 2018; Gómez-Ferrer et al., 2021). Third, the quantity and purity of native MSC-EVs derived from national MSCs frequently fail to meet the stringent requirements for clinical translation; however, the emerging 3D culture systems and physical stimulation strategies have demonstrated significant efficacy in enhancing EVs production while preserving their therapeutic quality (Yuan et al., 2022; Li S. et al., 2023). Fourth, engineered MSC-EVs can be tailored to overcome disease-specific pathophysiological barriers; for example, hypoxia-preconditioned MSC-EVs exhibit superior anti-apoptotic and pro-angiogenic activities that are particularly beneficial for ischemic tissue repair. Collectively, these engineering strategies transform MSC-EVs from passive biological messengers into programmable drug delivery platforms with enhanced efficacy, specificity, and reproducibility. However, natural MSC-EVs lack the ability for active targeting and rely on passive accumulation, resulting in limited efficacy. Engineered MSC-EVs achieve tissue-specific delivery through surface modification and can also carry therapeutic molecules (Meng et al., 2023). A growing body of evidence has suggested the engineering or pretreatment can enhance the proliferation, migration, directed differentiation, and anti-apoptotic properties of MSCs, thereby augmenting the secretion and functionality of MSC-EVs (Phan et al., 2018). The pretreated MSC-EVs have been demonstrated to enhance the anti-inflammatory and immunomodulatory effects, leading to improved therapeutic outcomes (Gómez-Ferrer et al., 2021; Pendse et al., 2021; Dong et al., 2021; Hou et al., 2024). There are several well-documented ways for the preconditioning of MSCs and MSC-EVs, such as pharmacological agents, hypoxic conditions, and genetic modifications. Common engineering methods are summarized in Figure 1.

### 3.1 Pharmacological intervention

Tanshinone IIA (TSA) has been identified as an efficacious therapeutic agent for myocardial ischemia/reperfusion injury (MI/RI). It has been demonstrated that miR-223-5p, delivering in MSC exosomes pretreated with TSA, can alleviate MI/RI by substantially promoting angiogenesis and reducing monocyte infiltration via CCR2 inactivation (Li S. et al., 2023). Another study has demonstrated that melatonin-pretreated MSC exosomes more effectively suppress the production of pro-inflammatory mediators of IL-1β, TNF-α and iNOS by activating the PTEN/AKT pathway, but significantly promote the generation of anti-inflammatory factors of IL-10 and Arg-1, thereby facilitating anti-inflammatory M2 polarization and wound healing in diabetic mice (Liu et al., 2020). Besides, BM-MSC-EVs pretreated with atorvastatin (ATV) exhibits enhanced cardiac repair capabilities in acute myocardial infarction (AMI) by increasing M2 macrophage polarization through the miR-139-3p/Stat1 signaling pathway (Ning et al., 2023). Furthermore, dexamethasone (Dex)-preconditioned MSC-EVs can reduce the expression of pro-inflammatory M1-associated genes but enhance the expression of anti-inflammatory M2-associated genes in LPS-stimulated macrophages (Mirsanei et al., 2024). Therefore, Dex-preconditioned MSC-EVs enhance the therapeutic efficacy by reversing the pro-inflammatory macrophages into anti-inflammatory macrophages. As an innovative drug delivery system, MSC exosomes is capable of transporting herbal extracts to the brains of Alzheimer’s disease mice, which alleviates Alzheimer’s disease symptoms by inducing autophagy and enhancing the cognitive and motor functions (Iyaswamy et al., 2023). Additionally, bevacizumab-loaded MSC-EVs are found to decrease the frequency of intravitreal injections required for diabetic retinopathy treatment, thereby reducing patient discomfort (Reddy et al., 2023). The doxorubicin-loaded BM-MSC exosomes (Exo-Dox) shows reduced cytotoxicity to H9C2 cardiomyocytes and increased antitumor activity in osteosarcoma (Wei et al., 2019). Modification of drugs encapsulated within EVs can enhance their pharmacokinetic and pharmacodynamic profiles (Böttger et al., 2020). Kartogenin (KGN) is a small molecule drug utilized in the treatment of osteoarthritis (OA) due to its effect on the inhibition of cartilage degeneration, which can promote the chondrogenic differentiation of synovial fluid-derived MSCs (SF-MSCs). However, its limited aqueous solubility restricts its chondrogenic efficacy. The fusion of the MSC-targeting peptide E7 with the exosomes membrane protein Lamp2b results in the formation of E7 exosomes (Xu et al., 2021). When co-administration with KGN via intra-articular injection in OA rat models, E7-exosomes exhibits significantly improved therapeutic outcomes compared to KGN administration alone or exosomes-delivering KGN without the E7 peptide (Xu et al., 2021). Taken together, the preconditioning of MSCs and MSC-EVs by pretreating or loading with various pharmacological agents can augment their anti-inflammatory, regenerative, and reparative capabilities, thereby providing improved therapeutic options for a variety of diseases, such as cardiovascular, neurological, and skeletal disorders.

### 3.2 Genetic modification

Gene transfection may introduce exogenous nucleic acid molecules, such as DNA, and RNA, into cells through physical, chemical or biological methods, enabling cells to express the proteins encoded by the exogenous genes or to achieve specific genetic functions. Research has demonstrated that glial cell line-derived neurotrophic factor (GDNF) augments the reparative effects of MSCs on renal injury (Chen et al., 2020). Exosomes derived from GDNF-transfected AT-MSCs significantly attenuate peritubular capillary (PTC) formation and inhibit renal fibrosis in mice with unilateral ureteral obstruction, primarily through the activation of the SIRT1/iNOS signaling pathway (Chen et al., 2020). CD38, highly expressed in hepatocellular carcinoma (HCC), can be effectively targeted by bone marrow MSC exosomes loaded with CD38 siRNA (Exo/siCD38). Exo/siCD38 has been found to suppress the growth and metastasis of HCC by inhibiting CD38 enzyme activity, reducing adenosine production, facilitating M1 macrophage repolarization, and reversing tumor resistance to PD-1/PD-L1 inhibitors (Deng and Ke, 2023). Similarly, MSC-EVs with overexpression of heat shock factor 1 (HSF1) exhibit enhanced protective effects against pulmonary injury caused by severe hemorrhagic shock (Jia et al., 2023). This is achieved primarily through significant inhibition of inflammatory cytokine production, neutrophil infiltration, and oxidative stress in the lung, and the maintenance of the pulmonary epithelial barrier integrity (Jia et al., 2023). Furthermore, the overexpression of microRNA-181a in MSCs markedly augments the therapeutic efficacy of MSC-EVs in treating ARDS-induced lung injury by reducing the secretion of TNF-α and IL-8 (Su et al., 2023). Additionally, bone marrow-derived MSC exosomes enriched with microRNA-29a promote bone regeneration by modulating osteogenesis and angiogenesis (Pan et al., 2024).

Due to high efficiency and targeting ability, the clustered regularly interspaced palindromic repeats/Cas9(CRISPR/Cas9) system is used to modify specific genes of MSCs, thereby reducing the immunogenicity of MSCs and enhancing their anti-inflammatory function (Park et al., 2024; Dashti et al., 2025). Compared with plasmid DNA, the CRISPR/Cas9 technology can achieve high insertion-deletion frequency and low cytotoxicity in MSCs for gene editing (Han HRS et al., 2024). After deleting the specific protein (SP1) in MSCs using CRISPR/Cas9, MSC-EVs exhibit the anti-apoptotic protective effect against renal ischemia-reperfusion injury, suggesting the high efficiency of this technology in editing the target gene (Yuan et al., 2017). The CRISPR technology is used to activate TSG-6 in MSCs, which enhances the expression of TSG-6 protein in MSC-EVs inhibiting inflammation (Martinez-Zalbidea et al., 2026; Martinez-Zalbidea et al., 2025). What is even more interesting is that MSC-EVs can effectively protect the CRISPR components from degradation due to their inherent delivery properties and modifiable design, thus becoming an excellent delivery vehicle for CRISPR therapeutic gene editing systems (Hazrati et al., 2022). MSC exosomes modified with chondrocyte affinity peptide (Cap) can specifically deliver the components of CRISPR/Cas9 into the chondrocytes of OA patients, thereby achieving precise and efficient ASPN gene knockout (Lou et al., 2026). The ^124^I@EVs-Cas9 constructed using BMSC-EVs can effectively inhibit the proliferation and metastasis of osteosarcoma by suppressing the expression of the inward-rectifying potassium channel subfamily J member 2 (KCNJ2) and promoting the ubiquitin-dependent degradation of HIF-1α (Pan et al., 2025). The tonsil-derived MSCs (TMSCs) overexpressed with TGF-β1 using the CRISPR/Cas9 system can better maintain the homeostasis of the cartilage microenvironment and protect the cartilage (Kim et al., 2024). Accordingly, with the innovation of biomanufacturing technologies and targeted strategies, engineered MSC-EVs are expected to provide a breakthrough solution for gene therapy (Whitley and Cai, 2023).

### 3.3 Hypoxic treatment

Hypoxia can trigger the self-protection mechanism of cells, leading to elevated production of anti-inflammatory, anti-apoptotic, and anti-fibrotic factors. It has been well established that hypoxic preconditioning can enhance the survival and metabolic activity of MSCs (Lan et al., 2015; Braga et al., 2022). Research has confirmed that MSCs with an oxygen concentration range of 1%–5% (physiological hypoxia) and a continuous short-term treatment (24–48 h) secrete more EVs that are enriched with molecules related to angiogenesis, and have stronger ability to repair tissue damage (Cavallero et al., 2023; Yang et al., 2022). Hypoxia markedly augments the pro-angiogenic properties of exosomes originating from olfactory mucosa MSCs (OM-MSCs) probably due to elevated levels of miR-612 within OM-MSC-EVs, which facilitates the angiogenesis via the miR-612-TP53-HIF-1α-VEGF signal axis (Ge et al., 2021). Ding et al. have demonstrated that apoptotic exosomes (H-Apo EVs) derived from the AT-MSCs under hypoxic conditions significantly enhanced the regenerative capacity of endogenous stem cells and M2 polarization of bone marrow-derived macrophages, thereby markedly promoting cartilage regeneration and repair (Ding et al., 2024). Therefore, the hypoxia-preconditioned MSC exosomes exhibit superior therapeutic efficacy in osteochondral regeneration by regulating MSC activity and the immune microenvironment (Ding et al., 2024). Hypoxia-preconditioned EVs (HS-EVs) restrain neuronal loss and attenuate spinal cord inflammation and injury by transferring miR-146a-5p, which induces macrophage M2 polarization and inhibits oxidative stress (Liang et al., 2024). Similarly, exosomes form hypoxia-preconditioned MSCs contribute to neuroprotection and facilitate the restoration of motor function following spinal cord injury via miR-21/JAK2/STAT3 signaling pathway (Yang et al., 2024).

Across the reviewed studies, hypoxia preconditioning conditions varied in oxygen concentration and duration. For instance, Ge et al. have found that the secreted EVs by subjecting OM-MSCs to 3% O_2_ for 48 h carried elevated levels of miR-612 and exhibited enhanced pro-angiogenic capacity (Ge et al., 2021). The apoptotic EVs from AT-MSCs cultured under hypoxic conditions exhibit superior chondroprotective activity compared with EVs from normoxic counterparts (Ding et al., 2024). More broadly, a comprehensive review of hypoxia-preconditioned MSC-EVs by Casado-Díaz et al. noted that most studies have employed O_2_ concentrations ranging from 0.1% to 5%, with treatment durations between 24 and 72 h (Pulido-Escribano et al., 2022). The hypoxia-preconditioned MSC-derived EVs possess a higher regenerative capacity than those obtained under normoxia, owing to the upregulation of genes associated with anti-apoptotic, pro-angiogenic, and immunomodulatory processes via HIF-1α induction (Pulido-Escribano et al., 2022). However, the optimal protocol of hypoxia-preconditioned MSC-EVs has not yet been reached, as the ideal hypoxia conditions may vary depending on MSCs source, target disease, and desired therapeutic outcome (Pulido-Escribano et al., 2022). Therefore, further systematic optimization in future studies is warranted to define standardized preconditioning parameters. In conclusion, currently available data have strongly supported that MSC-EVs derived from hypoxia-preconditioned MSCs exhibit higher anti-inflammatory, anti-apoptotic, pro-angiogenic, pro-regenerative, and homeostasis-maintaining properties compared to those obtained from untreated MSCs. The hypoxia-preconditioned MSC-EVs may serve as a promising approach for tissue regeneration, repair and immune homeostasis maintenance.

### 3.4 Cytokines priming

Preconditioned MSCs with pro-inflammatory cytokines has been demonstrated to not only promotes cell proliferation and survival but enhances the activity of MSC exosomes (Yao et al., 2021). The preconditioning of MSCs with TNF-α and/or IFN-γ significantly enhances the immunomodulatory effect of MSC-EVs (Kan et al., 2022). Specifically, under the synergistic effect of TNF-α and IFN-γ, MSCs promote RAB27B-regulated MSC-EVs generation, which deliver increased level of the immunomodulatory cargo RAB27B (Cheng et al., 2020). In comparison to exosomes derived from the unstimulated MSCs, exosomes from IL-1β-pretreated MSCs transfer higher level of miR-21, which significantly alleviates sepsis symptoms by facilitating M2-type macrophage polarization in a murine sepsis model (Bulati et al., 2020). Similarly, exosomes derived from IFN-γ-primed MSCs (IFN-γ exosomes) are enriched with miR-125a-5p, which play a regulatory role in both innate and adaptive immune responses (Yao et al., 2021). Another study has demonstrated that IFN-γ exosomes could effectively inhibit apoptosis and enhance angiogenesis due to the upregulation of miR-21 in IFN-γ exosomes, which improve the cardiac function following myocardial infarction through the STAT1/miR-21/BTG2 signaling pathway (Zhang et al., 2022). Additionally, BM-MSCs with TNF-α and IFN-γ priming can notably suppress the activation of natural killer and B lymphocytes by releasing exosomes encapsulating high levels of ICAM-1 and specific microRNAs (López-García and Castro-Manrreza, 2021). Upon stimulation with IFN-γ, MSC exosomes exhibit a marked upregulation of PD-L1, which subsequently attenuates CD4^+^ T cell activation via the PD-L1/PD-1 signaling pathway, thereby contributing to the amelioration of graft-versus-host disease (Li et al., 2021). Taken together, EVs-derived from cytokines-primed MSCs, such as TNF-α, IFN-γ, and IL-1β, possess enhanced activity in the regulation of inflammation and immunity by effectively delivering specific bioactive molecules, including miR-21, miR-125a-5p, and RAB27B.

MSCs have considerable potential for regenerative medicine, yet therapeutic effects remain inconsistent. Cytokine pretreatment represents one strategy to enhance their therapeutic potential (Valencia et al., 2025). Although cytokine priming can augment the immunomodulatory and tissue repair functions of MSC-EVs, it also carries potential risks that must be carefully considered. Pro-inflammatory cytokine stimulation particularly with TNF-α and IFN-γ can alter the secretory profile of MSCs and may upregulate certain pro-inflammatory mediators, potentially exacerbating inflammation in susceptible microenvironments (Soltero-Rivera et al., 2025). Furthermore, the possibility that cytokine-primed MSC-EVs may deliver undesired signaling molecules-such as pro-fibrotic factors or chemokines that recruit inflammatory cells to injury sites-cannot be excluded and warrants further investigation. Careful optimization of cytokine type, concentration, priming duration, and target disease context is therefore essential to maximize therapeutic efficacy while minimizing potential adverse effects.

With regard to the universal applicability of cytokine priming across different MSC sources, emerging evidence suggests that cytokine stimulation can reduce the inherent heterogeneity of resting MSCs from various tissue origins. Single-cell transcriptomic analyses have shown that TNF-α and IFN-γ priming induces a convergent immunomodulatory gene expression program in BM-MSCs, AT-MSCs, and UC-MSCs, effectively minimizing donor-to-donor and tissue-to-tissue variability (Wiese et al., 2022; Wan et al., 2023). This dynamic reprogramming effect makes cytokine priming a promising strategy for the development of standardized MSC-EVs products, particularly for allogeneic applications in which consistent potency is critical.

### 3.5 Hybrid EVs engineering strategies

Hybrid membrane nanovesicles (HMNVs) represent a class of advanced biomimetic nanocarriers engineered by integrating the inherent biological merits of natural EVs with the tunable physicochemical properties of synthetic nanovesicles. Endowed with exceptional biocompatibility, ligand-mediated targeting specificity, and high-efficiency payload encapsulation capacity, HMNVs effectively circumvent the critical limitations of conventional delivery systems, including suboptimal *in vivo* stability, restricted functional versatility, and off-target accumulation (Wang et al., 2025a; Zhang et al., 2025). Compared with LNPs, the EV-LNP mixture exhibits lower cytotoxicity (Ding et al., 2025). As a carrier, PLGA nanoparticles encapsulated the exosomes derived from human UC-MSC, forming a composite system with sustained-release properties. This enabled the sustained therapeutic concentration of hUC-MSC exosomes to be maintained at the site of bone resorption for a long time (Xie J. et al., 2023). The MSC-Hyb NPs constructed by microfluidic ultrasound technology can efficiently encapsulate type I collagen mRNA, and achieve functional mRNA delivery in tendon stem cells, breaking through the inherent drug loading capacity limitation of MSC-EVs and providing a new drug delivery platform for tendon regeneration (Pareja Tello et al., 2025). The mixed nanovesicles constructed by fusing liposomes modified with bone-muscle dual-targeting peptides and exosomes derived from induced pluripotent stem cells can precisely deliver miR-206-5p to target and inhibit the expression of DUSP4, activating the p38 MAPK pathway and promoting osteogenic and myogenic differentiation. These nanovesicles exhibit superior therapeutic effects in the mouse model of osteomuscular atrophy (Che et al., 2026). By using the ethanol-mediated fusion strategy to combine functional lipid nanoparticles with overexpressed TGF-β1 TMSC-EVs, Ab-Hybrid hybrid particles encapsulating nicotinamide and modified Col2A1 antibody were constructed. These particles demonstrated targeted and persistent chondroprotective and anti-inflammatory effects in an experimental osteoarthritis mouse model (Kim et al., 2024). Due to the significant differences in the hybrid vesicles produced by various fusion methods, a multi-index framework is required to evaluate the fusion efficiency, purity, and physical changes of the vesicles (Mizenko et al., 2026). As a cutting-edge nanotechnological platform, HMNVs have emerged as a transformative paradigm in translational medicine, holding immense potential for precision disease diagnosis, targeted therapeutic intervention, and personalized theranostic applications.

### 3.6 Other methods

#### 3.6.1 3D culture

The expansion of MSCs is essential for the acquisition of MSC-EVs (Hassan et al., 2020). Three-dimensional (3D) microcarrier culture systems more accurately mimic the physiological conditions of MSCs growth, thereby preserving their natural morphology and activity. Yuan et al. reported that human MSC-derived EVs increased by approximately twofold when cultured in 3D dynamic bioreactors compared to 2D static conditions, with altered protein cargo including upregulation of cytokines and anti-inflammatory factors (Yuan et al., 2022). This enhanced secretion has been attributed to the activation of both ESCRT-dependent and ESCRT-independent vesicle secretion pathways and cytoskeletal rearrangement in the 3D state (Yuan et al., 2022). Furthermore, 3D culture system can modify the composition of the cell membrane due to fluid shear stress. Metabolomic analysis has revealed that MSCs cultured in 3D bioreactors exhibit altered fatty acid and phospholipid compositions compared to those cultured in 2D conditions (Kurogi et al., 2022). These variations in lipid profiles may contribute to functional differences in MSC-EVs from different culture origins. Lee et al. (Lee et al., 2023) have found that MSCs cultured in a 3D system produced EVs enriched with microRNAs that facilitate the macrophage polarization towards M2, thereby effectively inhibiting inflammation in islets and preserving beta cells functions. Consequently, MSC-EVs obtained from the 3D culture system show superior advantages in both quantity and quality, serving as a better therapeutic application for diseases.

#### 3.6.2 Physical stimulation

Low-intensity pulsed ultrasound (LIPUS) as a non-invasive physical stimulation method can enhance the therapeutic potential of MSC-EVs by regulating their biological characteristics. LIPUS generates mechanical stimuli at the cellular level, which induce transient effects on the cell membrane and activate mechanosensitive pathways, ultimately enhancing the biogenesis and trafficking of multivesicular bodies toward the plasma membrane. LIPUS stimulation has been shown to lead to a 3.66-fold increase in the secretion of BMSC-EVs. Mechanistically, RNA-seq analysis revealed that miR-328-5p and miR-487b-3p were significantly upregulated in LIPUS-treated BMSC-EVs, and the suppression of the MAPK signaling pathway by these miRNAs could be the potential mechanism underlying the enhanced anti-inflammatory effects (Li X. et al., 2023). LIPUS has been documented as an effective technique for augmenting the production and functional efficacy of oral MSC-EVs. LIPUS stimulation of stem cells from the apical papilla (SCAP) enhances the expression of neutral sphingomyelinase, which promotes EVs secretion and the miR-935-loading capacity of SCAP-derived EVs (Zhang et al., 2023). The miR-935 encapsulated in SCAP-EVs facilitates osteogenic differentiation and exerts anti-inflammatory effects on periodontal ligament cells, thereby mitigating inflammatory bone loss within the oral cavity (Zhang et al., 2023). Similarly, a study by Wang et al. demonstrated that low-intensity ultrasound stimulation of chondrocytes resulted in a 16-fold increase in EVs secretion, and these ultrasound-stimulated chondrocyte-derived EVs were enriched in cartilage-regeneration-related miRNAs and exhibited superior efficacy in promoting the chondrogenic differentiation of adipose-derived stem cells (Wang et al., 2023). A comprehensive review by He et al. has further summarized the roles and mechanisms of LIPUS in regulating stem/progenitor cell-derived exosomes and non-coding RNAs, highlighting the mechanical, thermal, and cavitation effects of LIPUS that contribute to altered cellular microenvironments and enhanced exosome functions (He et al., 2023).

Beyond LIPUS, several other physical stimulation methods have been explored to augment MSC-EVs production and functionality. Mechanical stimulation via cyclic stretch or shear stress has been shown to enhance EVs secretion from MSCs by mimicking the mechanical cues experienced in native tissue environments; for example, exosomes derived from cyclic mechanical stretch-treated BMSCs have been demonstrated to inhibit osteoclastogenesis through the NF-κB signaling pathway (Xia et al., 2021). Electrical stimulation has also been reported to modulate the cargo and therapeutic function of MSC-EVs; a comprehensive review by Wu et al. on physical modulation of MSC exosomes documented that electrical stimulation increases both the yield and the therapeutic cargo of MSC-EVs (Wu et al., 2024). Photobiomodulation using low-level laser therapy has demonstrated effects on EV secretion and function from MSCs *in vitro*; for instance, 830 nm near-infrared laser irradiation was found to increase EVs yield from human adipose-derived MSCs by 6.25-fold at an optimal dose (Chang et al., 2024), and 660 nm irradiation was shown to modulate the structure and function of EVs secreted from periodontal ligament MSCs and adipose-derived MSCs (Gholami et al., 2022). A broader review on scalable EVs production has further summarized mechanical stimulation, electrical stimulation, and other physical strategies as effective approaches for enhancing EVs yield and modifying EVs cargo (Ng et al., 2022). To date, however, LIPUS remains the most extensively characterized physical stimulation method for MSC-EVs engineering due to its non-invasive nature, favorable safety profile, and ease of clinical translation (He et al., 2023).

## 4 Applications of MSC-EVs in clinical diseases: clinical safety considerations

With the advance of basic research and biological techniques, the clinical applications of MSC-EVs have become prevalent during the past few years. The core objective of these studies is to comprehensively evaluate the efficacy and safety of MSC-EVs in the treatment of various diseases, providing practical support for the safety of drug-loading in MSC-EVs in the future. This paper has summarized currently available studies on the use of MSC-EVs in different clinical diseases. Detailed information has been shown in Table 1.

**TABLE 1:** Summary of studies on MSC-EVs treatment in different clinical diseases.

Study subjects	Study design	Number of cases	Source of MSC-EVs	MSC-EVs dose	Usage/ dosage	Follow-up index	Reference
COVID-19 patients with ARDS	phase II randomized, multicentric clinical trial	43	BM-MSC-EVs	MSC-EVs (isolated from the 200 × 10^6^ ± 10% cells)	one dose of MSCs intravenously and one dose of MSC-EVs through inhalation route	clinical symptoms, laboratory parameters and inflammatory markers	Zarrabi et al. (2023)
Severe COVID-19 with moderate-to-severe ARDS	phase II multicenter, double-blind, randomized, placebo-controlled dosing trial	102	BM-MSC-EVs	15 mL ExoFlo group (1.2 × 10^12^ particles),10 mL ExoFlo group (0.9× 10^12^ particles)	intravenous infusion on day1 and day4	the mortality rate within 60 days, viremia, serum acute phase reactants, immune cell subset counts	Lightner et al. (2023)
Severe COVID-19 and moderate-to-severe ARDS patients	prospective nonrandomized open-label cohort study	24	BM-MSC exosomes	15 mL ExoFlo	a single intravenous	infusion reactions, adverse events, efficacy (including PaO2/FiO2, oxygen support requirements, degree of inflammation and immunocompetence)	Sengupta et al. (2020)
COVID-19 and ARDS	randomized, double-blind, placebo-controlled pilot trial	30	hUC-MSC exosomes	5 × 10^10^ particles	a single intravenous	clinical symptoms, laboratory parameters and inflammatory markers	Hosseinzadeh et al. (2025)
COVID-19-associated ARDS	double-blind randomized controlled clinical trial	21	hPMSC-sEVs	1.5–2 × 109 EVs per kilogram of body weight	cubital veinaccess for two consecutive days	SpO2, body temperature, erythrocyte sedimentation rate (ESR), D-dimer levels routine, blood tests	Zamanian et al. (2024)
COVID-19 pneumonia patients	pilot trial	7	UC-MSC exosomes	5 mL (7.0 × 10^7^–7.66 × 10^8^ particles/mL)	nebulization treatments were performed twice a day until discharge	secondary infection, allergic reactions, life-threatening adverse events, inflammatory cell count, chest CT	Chu et al. (2022)
Severe COVID-19- related pneumonia patients	phase 2a single-arm, open-labelled, interventional trial	7	AT-MSC-EVs	2.0 × 10^8^ nano vesicles	nebulization treatment continued for 5 days	inhalation-associated events and serious adverse events, lymphocyte count, levels of D-dimer and IL-6 as well as chest imaging	Zhu et al. (2022)
COVID-19 patients	double-blind, multicentered, randomized, placebo-controlled trial	20	MSC secretome	15 mL MSC secretome	intravenous administration	inflammatory markers, clinical outcome, laboratory outcome, radiological outcome	Abdullah et al. (2022)
COPD patients	cohort study	30	placental tissue MSC exosomes	0.5mLexo-d-MAPPS (exosomes, growth factors, and immunomodulatory cytokines)	nebulization treatment once per week for 3 weeks	spirometry, chest CT, standard clinical COPD questionnaire (CCQ) scoring, and 6-min walking distance (6MWD) test	Harrell et al. (2020)
Pulmonary fibrosis patients	randomized, single-blind, and placebo-controlled study	24	hUC-MSC-EVs	2 mL of hUC-MSC-EVs (2 × 109particles)	the nebulization treatment is administered twice a day for a total of 7 days	lung function indexes, HRCT images	Li et al. (2025)
Healthy volunteers	phase single-arm clinical trial	24	AT-MSC-EVs	first cohort (2.0 × 10^8^ particles), second cohort (4.0 × 10^8^ particles), third cohort (8.0 × 10^8^ particles), fourth cohort (12.0 × 10^8^ particles), fifth cohort (16.0 × 10^8^ particles)	a single nebulization	vital sign (temperature, heart rate, respiratory rate and saturation oxygen), laboratory tests such as blood routine test, liver and renal function, lactate dehydrogenase, immunoglobulin	Shi et al. (2021)
Healthy volunteers	phase 1, open-label study	10	MSC exosomes	100 mg	apply to the healthy skin area of the forearm for 20 days, three times a day, with an interval of 4 h each time	clinical laboratory evaluation, adverse events	Chandran et al. (2025)
Facial skin aging	prospective, randomized, split-face study	28	AT-MSC exosomes	5 × 10^9^ particles	microneedling, three treatment sessions separated by 3-week	surface roughness of the skin, skin biopsies	Park et al. (2023)
Facial skin aging	investigator-blinded, split-face trial	15	AT-MSC exosomes	20 mg	full-face RFMN treatments, three treatment sessions separated by 4-week	overall skin appearance, wrinkling, dyschromia, erythema	Estupiñan et al. (2025)
Skin aging	randomized, double-blinded, placebo-controlled study	40	perinatal MSC exosomes	5 × 10^9^ exosomes	microneedling, once every 30 days for a total of four times	allergic reactions, adverse events, the 3D Quantificare analysis, patient satisfaction	Chernoff (2021)
Skin aging	randomized controlled trial	40	perinatal MSC exosomes	1 × 10^9^ exosomes	a single microneedling	allergic reactions, adverse events, the 3D Quantificare analysis, patient satisfaction	Chernoff (2023)
AD patients and healthy volunteers	a single-arm study	7	WJ-MSC-EVs	42 µg (AD patients) 210 µg (healthy volunteers)	patch test for 5 days (AD patients) apply for 15 days (healthy volunteers)	clinical evaluations and patient-reported symptoms	Elgueta et al. (2025)
AD patients and refractory dupilumab facial redness	case report	2	AT-MSC exosomes	2.0 × 10^9^ particles	microneedling, six repeated sessions of treatment, with an interval of 1 week between each session	clinical examination, patient satisfaction	Park et al. (2022)
AD patients	cohort study	28	conditioned media from UC-MSCs	unspecified	topical application, twice a day for 4 weeks	corneometer, TEWL levels	Kim et al. (2020)
Acne, mild acne scarring and lip trauma patients	case report	2	placental tissue MSC exosomes	acne, mild acne scarring (1.5 × 10^10^ exosomes), lip trauma (12.5 × 10^9^ exosomes)	a single topical application	clinical examination, patient satisfaction	Peredo and Shivananjappa (2024)
Recurrent ischial ulcer patients	case report	1	MSC exosomes	1 mL exosomes	six subcutaneous exosomes injections over 8 weeks	clinical examination	Messa et al. (2022)
DFUs	randomized double-blind controlled clinical experiment	110	WJ-MSC exosomes	unknown	applied to wounds once weekly for 4 weeks	imaging studies and laboratory tests	Kishta et al. (2025)
DFUs	single-center, phase I/II, open-label clinical trial	10	hUC-MSCD	5 mL	injections at the edge of the ulcer, once a week for a total of 10 times	laboratory tests and clinical examination	Jafar et al. (2025)
Chronic cutaneous GVHD	case report	1	placental MSC exosomes	1.9–2.6 × 10^11^ particles	intravenous injection, four treatments at a weekly interval	clinical examination, inflammatory cell counts, liver function tests	Norooznezhad et al. (2022)
Sensitive skin	cohort study	22	UC-MSC exosomes	1 mL exosomes	twice a day	TEWL, surface hydration, sebum secretion, patient satisfaction	Ye et al. (2022)
Mild to moderate AD patients	three-arm, open-label, single-center, drug-intervention, phase I/II clinical trial	9	AT-MSC exosomes	low-dose group (2 × 10^8^ particles), middle-dose group (4 × 10^8^ particles), high-dose group (8 × 10^8^ particles)	intranasally administrated with AT-MSC exosomes two times per week for 12 weeks	adverse events, Alzheimer’s Disease Assessment Scale–Cognitive section (ADAS-cog) scores, amyloid or tau deposition	Xie X. et al. (2023)
Decompressive craniectomy following malignant middle cerebral artery infarct	pilot randomized clinical trial	5	Placental MSC exosomes	2 mL (356 μg/mL)	a single dose-intraparenchymal injection	safety assessments and neurological status	Dehghani et al. (2022)
Traumatic brain injury patients	phase I study	5	MSC exosomes	30 billion exosomes	the treatment is conducted once every 4 weeks for a total of six times (3 mL are injected intrathecally and 3 mL are administered by intramuscular injection in each session)	MAS, FIM and KPS scales, neuropathic pain, urinary tract infections, secondary infections, and pressure ulcers	Kabatas et al. (2025)
Spinal cord injury patients	single-arm, open-label, phase I clinical trial	9	hUC-MSC exosomes	unknown	intrathecal injection	adverse event, neurological assessment, functional assessment	Akhlaghpasand et al. (2024)
Total radial nerve injury patients	case report	1	WJ-MSC exosomes	5 ×10^7^ microvesicles	a single local microinjection	Mackinnon-Dellon scale, electromyography	Civelek et al. (2024)
Large and refractory macular holes patients	cohort study	5	UC-MSC exosomes	50 μg or 20 μg	a single intraocular drip	BCVA measurements, fundoscopy, OCT, physical examinations	Zhang Y. et al. (2018)
GVHD-associated dry eye disease patients	cohort study	14	UC-MSC exosomes	10ug/50ul	intraocular medication four times a day for 2 weeks.	clinical examination, ocular surface disease index scores	Zhou et al. (2022)
Refractory perianal fistula in IBD patients	open-label, phase I prospective clinical trial study	5	UC-MSC exosomes	5 mL (50ug/mL)	a single local injection	clinical examination, complete blood count, liver function tests, erythrocyte sedimen-tation rate, renal function tests	Nazari et al. (2022)
Perianal fistulain patients	clinical trial phase I	11	placental MSC exosomes	5 mL (50ug/mL)	a single local injection	physical examination, face-to-face interviews, and magnetic resonance imaging	Pak et al. (2023)
CKD patients	phase II/III clinical pilot study	40	hUC-MSC-EVs	100 μg/kg	a single itra-arterial and intravenous injections with 1 week apart	renal function tests, TNF-α, TGF-β1, IL-10	Nassar et al. (2016)
OA	randomized, double-blind, dose-escalation clinical trial	41	hUC-MSC exosomes	low-dose group (3 × 10^11^ particles) mid-dose group (4 × 10^11^ particles) high-dose group (5 × 10^11^ particles)	intra-articular injection once every 21 days, for a total of 2 times	physical examinations, laboratory tests, and WOMAC scores	Wang et al. (2025b)
OA	randomized, triple-blind, placebo-controlled clinical trial	31	placental MSC-EVs	3.5 × 10^10^ particles	a single intra-articular injection	clinical symptoms or MRI findings	Bolandnazar et al. (2024)
Traumatic hemorrhage patients	double-blind, randomized, self-controlled, single-dose pilot study	39	MSC-apoVs	MSC-apoV gelatin sponge (4 × 10^10^ particles)	a local application	routine blood tests, liver and kidney function tests, bleeding time, Cone Beam Computed Tomography	Jiang et al. (2025)

hUC-MSCD, human umbilical cord mesenchymal stem cell derivatives; hPMSC-sEVs, human placental mesenchymal stromal cell-derived small extracellular vesicles; MSC-apoVs, MSC-derived apoptotic vesicles.

### 4.1 Pulmonary diseases

In clinical trials of pulmonary diseases assessing the safety and efficacy of MSC-EVs, MSC-EVs are predominantly obtained from adipose tissue, bone marrow, and perinatal tissues. The main methods for administration include intravenous injection and aerosol inhalation. Recent studies have suggested ARDS and multi-organ failure are the leading causes of mortality in patients with Coronavirus Disease 2019 (COVID-19). BM-MSCs treatment combining with MSC-EVs has been shown to significantly decrease serum inflammatory cytokines in COVID-19 patients without serious adverse events (Zarrabi et al., 2023). A prospective phase 2 multicenter trial has demonstrated that BM-MSC-EVs (ExoFlo) are safe for patients with severe COVID-19-related respiratory failure, effectively increasing ventilator-free days and reducing mortality (Lightner et al., 2023). Patients suffering from severe COVID-19 and moderate to severe ARDS exhibited marked clinical improvement and enhanced oxygenation following a single administration of BM-MSC exosomes or hUC-MSC exosomes (Sengupta et al., 2020; Hosseinzadeh et al., 2025). After continuous intravenous infusion of human placental mesenchymal stromal cell-derived small extracellular vesicles (hPMSC-sEVs) for 2 days, it can significantly reduce the mortality rate of patients with COVID-19-associated ARDS. Notably, this 2024 double-blind, randomized, placebo-controlled clinical trial represents one of the most rigorous evaluations to date, employing strict randomization and blinding protocols and demonstrating improved oxygenation indices without any serious adverse events attributable to the treatment (Zamanian et al., 2024). Furthermore, the aerosol inhalation of MSC-EVs facilitated the resolution of pulmonary lesions and reduced the duration of hospitalization among mild COVID-19 pneumonia patients, but did not cause acute or secondary allergic reactions (Chu et al., 2022). Following five consecutive days of aerosol inhalation of AT-MSC-EVs, no adverse events were reported in patients suffering from severe COVID-19 pneumonia, whereas lung lesions showed different degrees of regression (Zhu et al., 2022). Similarly, after intravenous injection of MSC secretome, the inflammatory levels in COVID-19 patients did not increase any further, and there were no adverse reactions (Abdullah et al., 2022). Chronic obstructive pulmonary disease (COPD) is a chronic respiratory condition characterized by high morbidity and mortality. It has been found the inhalation of exosomes derived from human placental MSCs can mitigate inflammation and emphysema among COPD patients, thereby significantly enhancing their quality of life (Harrell et al., 2020). Pulmonary fibrosis is a chronic progressive disease characterized by abnormal scarring of lung tissue. It significantly affects the quality of life of patients and can even lead to death. A 2025 phase I/II study investigating nebulized hUC-MSC-EVs in 24 patients with idiopathic pulmonary fibrosis reported significant improvements in forced vital capacity and 6-min walk distance after a 7-day treatment course, with no dose-limiting toxicities observed (Li et al., 2025). These recent large-cohort, randomized studies collectively reinforce the safety profile and therapeutic potential of MSC-EVs in pulmonary diseases. The safety of AT-MSC-EVs aerosol inhalation has also been preliminarily validated in healthy volunteers, which suggested MSC-EVs might be a promising therapeutic strategy for pulmonary diseases (Shi et al., 2021). All these studies have suggested the significant clinical value of MSC-EVs in treating pulmonary diseases.

As a result, MSC-EVs serve as a promising cell-free therapeutic strategy for some pulmonary diseases, such as COVID-19 and COPD. Although MSC-EVs with different origins show similar potentials in defending against lung lesions and cytokines storm, there are still limitations in some clinical studies, such as small sample size, individual variability, drug use method and dose, and the concurrent administration of other medications. These terms may influence the definitive evaluation of MSC-EVs treatment in pulmonary diseases. Thus, more high-quality clinical trials with larger sample sizes and extended follow-up time should be conducted in the future to more rigorously delineate the dosing, administration, efficacy, and safety profiles of MSC-EVs when treating different lung diseases.

### 4.2 Dermatological diseases

Alterations in the skin is one of the most common manifestations during aging. A 20-day study on the local application of MSC exosomes on the skin of 10 healthy adult volunteers showed that the tolerance was good and there were no serious adverse events (Chandran et al., 2025). Following three sessions of treatment with AT-MSC-derived exosomes (AT-MSC exosomes), there was a remarkable remission in skin wrinkles, elasticity, hydration, and pigmentation (Park et al., 2023; Estupiñan et al., 2025). Similarly, AT-MSC exosomes represents a safe and efficacious intervention for facial skin aging in a previous study (Chernoff, 2021). In a randomized, double-blind clinical trial involving participants with skin aging, MSC exosomes treatment has been demonstrated to significantly improve the skin quality, characterized by a reduction in wrinkles and pore size (Chernoff, 2021). Furthermore, MSC exosomes can be utilized independently or in combination with adjunctive agents, such as botulinum toxin, to enhance the therapeutic outcomes (Chernoff, 2023). Atopic dermatitis (AD) is characterized as a chronic, pruritic, and inflammatory dermatological disease. After three AD patients and four healthy volunteers received EVs derived from Wharton’s Jelly-MSC (WJ-MSC), no adverse local or systemic reactions were observed (Elgueta et al., 2025). Dupilumab, a monoclonal antibody targeting IL-4α, has been established as an effective drug for AD. Nevertheless, a subset of patients receiving Dupilumab therapy experience the side effect of facial eczematous rashes, whereas the localized application of AT-MSC exosomes via electroporation significantly ameliorates Dupilumab-induced refractory facial rashes in AD patients, with a notable reduction in facial erythema (Park et al., 2022). Furthermore, the conditioned medium derived from human umbilical cord blood MSCs (USC-CM) is enriched in anti-inflammatory factors, which can improve the skin barrier function of mild AD patients as evidenced by elevated corneometer readings and reduced transepidermal water loss (Kim et al., 2020). MSC exosomes from human placenta-derived MSCs have been shown to significantly ameliorate acne and erythema, expedite the healing process of traumatic wounds, and improve patients’ satisfaction with medical and aesthetic treatments (Peredo and Shivananjappa, 2024). In individuals with chronic pressure ulcers, the subcutaneous injection of MSC exosomes resulted in complete ulcer resolution (Messa et al., 2022). The treatment of diabetic foot ulcers (DFUs) is a clinical challenge. More recently, a 2025 randomized, double-blind, controlled clinical trial involving 110 patients with diabetic foot ulcers demonstrated that weekly topical application of WJ-MSC-derived exosomes for 4 weeks significantly accelerated wound closure rates and reduced ulcer area compared to standard care alone, with no treatment-related adverse events (Kishta et al., 2025). Additionally, a 2025 phase I/II trial evaluating human umbilical cord MSC derivatives in 10 patients with chronic DFUs confirmed the safety and tolerability of repeated perilesional injections over 10 weeks, with promising signals of efficacy (Jafar et al., 2025). These trials provide robust clinical evidence supporting the translational potential of MSC-EVs in chronic wound management. A previous study has documented that the administration of intravenous human placenta-derived MSC exosomes led to a marked reduction in skin inflammation, particularly in pigmentation and dryness associated with skin ulcers in a patient with acute myeloid leukemia (AML) subtype M4 who developed cutaneous Graft-versus-host disease (GVHD) following allogeneic peripheral blood stem cell transplantation (PBSCT) (Norooznezhad et al., 2022). Due to their biocompatibility and biodegradability, MSC exosomes have the potentials to ameliorate clinical symptoms and rashes in individuals with sensitive skin, suggesting MSC exosomes as a novel therapeutic approach for sensitive skin conditions (Ye et al., 2022). Increasing evidence has suggested AT-MSC-EVs are being increasingly utilized in the treatment of dermatological conditions because of the robust differentiation potential of AT-MSCs into adipocytes and epithelial cells responsible for skin structure and quality (Bunnell, 2021). Taken together, AT-MSC-EVs may be a promising treatment for dermatological diseases due to the acceptability and applicability in dermatological therapies. As a prevalent cosmetic procedure, liposuction facilitates the extraction of AT, which can be efficiently repurposed to obtain substantial quantities of AT-MSC-EVs.

In conclusion, MSC-EVs, including MSC exosomes, represents a promising therapeutic strategy for the treatment of dermatological diseases, such as AD, pressure ulcers, and acne. Notably, AT-MSCs exhibit an enhanced capacity to facilitate the regeneration of adipocytes and epithelial cells, potentially rendering them more effective in promoting skin tissue regeneration and repair.

### 4.3 Neurological diseases

A phase I/II clinical trial has provided evidence that the intranasal delivery of AT-MSC exosomes for the treatment of mild to moderate Alzheimer’s disease is both safe and well-tolerated (Xie X. et al., 2023). The cognitive function was notably improved among Alzheimer’s disease patients in the medium-dose cohort (4 × 10^8^ particles/mL). It has been demonstrated that the intracerebral implantation of MSC exosomes in patients with ischemic stroke did not result in any adverse effects, suggesting the administration of MSC exosomes in neurological diseases. Besides, allogenic placenta MSC exosomes hold promise as a supportive, restorative, and preventive intervention for acute ischemic stroke and post-ischemic disability (Dehghani et al., 2022). Brain injuries and spinal cord injuries both fall under severe neurological impairments, which may result in permanent functional disabilities and even endanger life. A phase I clinical trial has shown that MSC exosomes treatment is safe and effective in improving the motor function, cognitive ability and quality of life of patients with traumatic brain injury (Kabatas et al., 2025). Similarly, intrathecal injection of hUC-MSC exosomes is safe for patients with spinal cord injury, and it may be associated with clinical functional improvement (Akhlaghpasand et al., 2024). Moreover, another study has implicated that treatment with MSC exosomes facilitated the recovery of sensory and motor functions in a patient with left radial nerve injury, also resulting in a favorable prognosis (Civelek et al., 2024). It should be noted that clinical trials of MSC-EVs in neurological disorders remain predominantly in the early phase (Phase I), with small sample sizes and limited statistical power. The absence of large-scale Phase II/III trials for stroke, spinal cord injury, and other neurological conditions represents a critical gap that must be addressed to validate the translational potential of MSC-EVs in the neurological field. Consequently, MSC exosomes serves as a therapeutic option for neurological disorders, including stroke and Alzheimer’s disease. Nonetheless, further clinical evidence is required to comprehensively assess the efficacy and safety of MSC-EVs, beyond MSC exosomes, in the treatment of neurological diseases.

### 4.4 Ocular diseases

The administration of intravitreal MSC exosomes following vitrectomy in patients with refractory macular holes (MHs) is demonstrated to significantly enhance the recovery of both visual function and anatomical structure, thereby improving postoperative visual outcomes (Zhang X. et al., 2018). Dry eye disease associated with GVHD is marked by substantial inflammatory damages to the ocular surface, resulting in severe pain and visual impairment. In a prospective clinical trial, patients suffering from GVHD-associated refractory dry eye have got significant alleviation in both pain and visual impairment following BM-MSC exosomes treatment, underscoring the efficacy of MSC exosomes in managing GVHD-related dry eye (Zhou et al., 2022). Mechanistically, BM-MSC exosomes exert the therapeutic effects through the delivery of miR-204, which targets the IL-6/IL-6R/Stat3 signaling pathway and facilitates the reprogramming of pro-inflammatory M1 macrophages into anti-inflammatory M2 macrophages (Zhou et al., 2022). These studies have offered robust evidence supporting the clinical utility of MSC exosomes in treating ocular diseases. More future studies are warranted to estimate the role of MSC-EVs in biomolecules delivery, not merely BM-MSC exosomes, in ocular diseases.

### 4.5 Gastrointestinal fistulas

In a cohort study, three of the five patients with refractory perianal Crohn’s disease-associated fistulas have got complete healing following the intrafistular administration of MSC exosomes without obvious adverse effects (Nazari et al., 2022). Similarly, 5 of the 11 patients with complex perianal fistulas have achieved complete rehabilitation following the intrafistular injection of BM-MSC exosomes without any allergic reactions or other complications (Pak et al., 2023). These findings have suggested the potential of BM-MSC exosomes as a therapeutic approach in treating gastrointestinal fistulas. Nonetheless, additional high-quality clinical studies are necessary to thoroughly assess the clinical efficacy and safety of MSC exosomes and other MSC-EVs in the treatment of gastrointestinal fistulas in the future.

### 4.6 Renal diseases

A previous study has supported that the administration of MSC-EVs in patients with stage III and IV chronic kidney disease (CKD) led to significant improvement of clinical indicators, including blood urea, serum creatinine, urine albumin-to-creatinine ratio, and glomerular filtration rate (Nassar et al., 2016). Besides, there is a marked increase of plasma anti-inflammatory cytokines levels, including TGF-β1 and IL-10, but a significant reduction in the pro-inflammatory cytokine TNF-α among CKD patients receiving MSC-EVs treatment (Nassar et al., 2016). As a result, MSC-EVs are a promising therapeutic approach for CKD by mitigating inflammation and improving renal function. Nonetheless, there is the clinical research investigating the application of MSC-EVs in CKD remains limited. More future studies are needed to confirm the clinical efficacy and safety in treating CKD.

### 4.7 Bone disease

Osteoarthritis (OA) is a common chronic joint disorder, and it is one of the main causes of disability worldwide. Studies have shown that low, medium and high doses of intra-articular injections of hUC-MSC exosomes did not cause any adverse effects and could also alleviate the inflammatory response of the joint cartilage (Wang et al., 2025a). Another study has shown that a single intra-articular injection of placental MSC-EVs is safe and reliable, but it does not improve the clinical symptoms or MRI manifestations of knee OA (Bolandnazar et al., 2024). The uncontrollable bleeding and tissue damage caused by trauma are serious problems in clinical practice. Local application of freeze-dried MSC apoptotic vesicles (MSC-apoVs) can shorten the bleeding time and accelerate the speed of alveolar bone regeneration. This demonstrates that MSC-apoVs is a safe, well-tolerated and effective cell-free biological therapy in terms of hemostasis and bone regeneration (Jiang et al., 2025).

MSC-EVs, as a drug-carrying carrier, have been preliminarily verified for its safety in clinical treatment, including local and systemic application safety and dose-dependent safety. However, to fully assess its clinical application potential, more high-quality, large-sample, and long-term follow-up clinical trials are needed in the future to comprehensively evaluate the long-term safety and individual differences of MSC-EVs.

## 5 Challenges and prospects

In conclusion, utilizing MSC-EVs as drug delivery carriers has opened up a highly promising new avenue for the treatment of a variety of diseases. Their applications extensively cover fields such as pulmonary, dermatological, neurological, ocular, and gastrointestinal diseases. Leveraging their unique structure and function, MSC-EVs play a pivotal role in the process of biomolecule delivery. They can effectively load and transport drug molecules to target sites, demonstrating immense potential in disease treatment, precise drug delivery, and regenerative medicine. Through engineering approaches including drug intervention, hypoxia treatment, gene editing modification, and cytokine activation, the biological effects of MSC-EVs can be further enhanced, optimizing their clinical application performance as drug delivery carriers.

### 5.1 Comparison with other nanocarriers

In contrast to synthetic nanocarriers such as liposomes and polymeric nanoparticles, MSC-EVs possess inherent advantages that make them uniquely suited for drug delivery applications. Their natural phospholipid bilayer structure and surface protein repertoire confer low immunogenicity, high biocompatibility, and the ability to traverse biological barriers including the blood-brain barrier (Thakur et al., 2020a; Thakur et al., 2020b). Unlike synthetic carriers, MSC-EVs are enriched with bioactive molecules—including proteins, lipids, and nucleic acids-that may exert intrinsic therapeutic effects independently of the loaded drug cargo (Kesidou et al., 2024; Chen et al., 2019). However, synthetic nanocarriers currently offer superior batch-to-batch consistency, well-established large-scale manufacturing protocols, and greater flexibility in surface functionalization for active targeting (Zhang et al., 2025). Liposomes, for instance, benefit from decades of clinical experience and defined regulatory pathways, whereas polymeric nanoparticles allow precise control over drug release kinetics through tunable polymer chemistry (Gudbergsson et al., 2019). Compared to these platforms, MSC-EVs face challenges in standardization, purity, and yield, which must be addressed through advances in 3D culture systems, engineered cell lines, and improved purification technologies (Vader et al., 2016). A particularly promising direction is the development of hybrid EV-nanoparticle systems that combine the biocompatibility of natural EVs with the engineering precision of synthetic carriers, as discussed in Section 3.5. The key characteristics of different drug delivery platforms are summarized in Table 2.

**TABLE 2:** Comparison of MSC-EVs with other nanocarriers for drug delivery.

Feature	Biocompatibility	Immunogenicity	Blood-brain barrier penetration	Intrinsic therapeutic activity	Batch consistency	Scalability	Active targeting	Drug loading capacity	Regulatory pathway	Key references
MSC-EVs	High (natural origin)	Low	Inherent	Yes (proteins, miRNAs, lipids)	Variable	Emerging (3D culture, bioreactors)	Achievable via genetic engineering	Low to moderate	Evolving (ATMP framework)	Kesidou et al. (2024), Chen et al. (2019), Meng et al. (2023), Whitley and Cai (2023)
Liposomes	Moderate to high	Low to moderate	Limited	None	High	Established	Surface modification	High	Well-established	Zhang et al. (2025), Wang X. et al. (2025)
Polymeric nanoparticles	Variable	Variable	Limited	None	High	Established	Surface modification	High	Established	Zhang et al. (2025), Wang X. et al. (2025)
Lipid nanoparticles (LNPs)	Moderate	Moderate	Limited	None	High	Established	Surface modification	High	Emerging (EMA/FDA approved)	Ding et al. (2025)

### 5.2 Pharmacokinetic considerations

The pharmacokinetic profile of MSC-EVs represents a critical determinant of their therapeutic efficacy and an area requiring further elucidation. A comprehensive review by Gupta et al. has summarized recent findings in EVs *in vivo* pharmacokinetics, noting that systemically administered EVs exhibit rapid clearance from the circulation and that the liver and spleen are the predominant sites of EVs accumulation following intravenous injection, largely due to phagocytic uptake by resident macrophages. The same review highlights that EVs biodistribution is influenced by several key variables, including the route of administration, the cellular source and culture conditions used for EVs production, the isolation and purification methods employed, and the surface protein composition of the EVs themselves. Furthermore, advanced labeling and tracking techniques, including fluorescent proteins, lipophilic dyes, and radioisotope labeling, have been developed to monitor EVs fate *in vivo* and have provided valuable insights into their spatiotemporal distribution patterns (Gupta et al., 2023). A systematic review by Kang et al. has further demonstrated that EVs administered intravenously are rapidly distributed to the liver, spleen, lungs, and kidneys, with detection peaking in the liver and kidney within the first hour and in the lungs and spleen between 2 and 12 h post-administratio (Kang et al., 2021). Strategies to extend the circulation time of MSC-EVs, such as surface PEGylation to reduce immunogenicity, CD47 overexpression to evade phagocytic clearance, or encapsulation in hydrogels for sustained local release, have shown promise in preclinical models (Xia et al., 2024; Wei et al., 2021). Additionally, the route of administration significantly influences EVs biodistribution; for instance, intranasal delivery facilitates preferential brain accumulation, while inhalation targets pulmonary tissues, and local injection enables sustained retention at the disease site (Ren et al., 2025). Understanding and optimizing the pharmacokinetic properties of MSC-EVs will be essential for their successful translation as drug delivery carriers.

### 5.3 Current challenges

However, clinical trials on MSC-EVs as drug delivery carriers are currently insufficient, primarily due to their relatively limited disease spectrum. Existing researches have predominantly focused on the treatment of COVID-19-related pneumonia and dermatological diseases, with most studies still in the early stages of clinical trials. A 2025 systematic review of published clinical studies using cell-derived EVs identified 25 clinical trials, among which COVID-19/ARDS was the most frequently studied disease category (8 studies, 32%), followed by wound healing (5 studies, 20%); notably, only 7 of these studies (28%) were controlled trials, and the individual patient data meta-analysis of controlled COVID-19/ARDS trials revealed a significantly reduced mortality odds ratio of 0.46 (95% CI 0.26–0.81) for MSC-EVs interventions (Duong et al., 2026).

In the neurological field, clinical translation has been particularly limited. A 2025 review on MSC and MSC-EVs therapies for neurological disorders noted that while MSCs have shown promising results in preclinical models of traumatic brain injury, ischemic stroke, and spinal cord injury, there remains a conspicuous absence of large-scale Phase II/III clinical trials to validate the translational potential of MSC-EVs in these conditions (Yarahmadi et al., 2025). This observation is reinforced by a 2025 systematic analysis of global MSC-EV clinical trials, which reported that as of 2024, relatively few registered trials targeted neurological disorders and that most were still in Phase I, underscoring the critical gap that must be addressed (Wang et al., 2025b).

There is an urgent need for large-scale, long-term studies to comprehensively validate their safety and efficacy. As an emerging drug delivery system, the application of MSC-EVs faces numerous challenges. The foremost challenge lies in the high degree of heterogeneity of MSC-EVs, which is manifested in significant differences in their origin, size, contents, and even functions. The formation of MSC-EVs is a complex biological process regulated by multiple factors, including intracellular signaling pathways and environmental conditions. Simultaneously, MSCs themselves also exhibit marked heterogeneity, influenced by factors such as donor differences, tissue sources, and expansion passages, further exacerbating the diversity in the phenotypes and functions of MSC-EVs. This heterogeneity poses issues of low efficiency and poor reproducibility in both basic research and clinical applications (Yin J. Q. et al., 2019). Secondly, there is a lack of unified standards for the preparation and purification methods of MSC-EVs, resulting in inconsistent quantities, qualities, and functions of the vesicles. Impurity contamination during the preparation process may also reduce the purity of MSC-EVs, thereby affecting their therapeutic efficacy. Moreover, the regulatory mechanisms of MSC-EVs are not yet fully understood. Despite their low immunogenicity, MSC-EVs may still trigger certain immune and inflammatory responses. Studies using experimental models and samples from rheumatoid arthritis patients have confirmed that EVs can serve as carriers for certain pro-inflammatory mediators, such as microRNAs (Shaikh et al., 2024). Furthermore, the distribution and metabolism of MSC-EVs in the human body remain incompletely characterized, and the optimal dosage and frequency of administration have yet to be determined, all of which require further clinical research to establish. Additionally, the storage and transportation of MSC-EVs present significant logistical challenges, particularly as maintaining their stability and bioactivity necessitates strict low-temperature and sterile conditions, which substantially increase the costs associated with their clinical application and large-scale manufacturing.

### 5.4 Future directions and regulatory outlook

To address these challenges, future research should focus on optimizing the delivery routes of MSC-EVs based on disease characteristics and their pharmacokinetic profiles. Current known techniques, such as nebulized inhalation, microneedle therapy, and local injection, hold promise for enhancing the therapeutic effects of MSC-EVs. Furthermore, future clinical trials should aim to conduct large-scale, multicenter, and randomized controlled studies to broaden the application scope of MSC-EVs as drug delivery carriers and promote their widespread use in clinical treatment.

As a clinical drug, MSC-EVs need to clearly define their cellular origin, lipid membrane vesicle structure, and physicochemical integrity, and establish quantifiable biomarkers (such as specific protein profiles) to confirm identity and purity. Currently, there is a lack of a globally unified definition of MSC-EVs and quantitative standards for key characteristics (van Balkom et al., 2019). A 2024 review by Wang et al. systematically examined the global regulatory landscape of exosomes as biologic medicines, highlighting the chemistry, manufacturing, and control (CMC) requirements imposed across different jurisdictions, as well as the inherent challenges of demonstrating pharmacokinetics and therapeutic efficacy to regulatory agencies (Wang C. K. et al., 2024). A subsequent 2025 review on worldwide EVs-based drug development guidelines further noted that regulatory authorities in the European Union, the United States, and China are actively advancing technical frameworks to address the unique complexities of EVs-based products (Xu et al., 2025). The European Union follows the framework for advanced therapeutic medicinal products (ATMP) and conducts evaluations in accordance with the regulatory requirements for cell therapy products. Guided by the relevant regulations of The European Union Regulation 1394/2007, it focuses on aspects such as the production process, quality control, non-clinical research, and clinical research of EVs drugs (Varderidou-Minasian and Lorenowicz, 2020; Joyce et al., 2023). The FDA of the United States tends to regulate based on biological products, emphasizing the quality of CMC data and the IND application, and focusing on the safety, efficacy and quality controllability of EVs drugs (Mitrani et al., 2021). The National Medical Products Administration of China has incorporated MSC-EVs into the ATMP framework and implemented classified management, emphasizing full life-cycle control (Zhang et al., 2024; Yuan et al., 2025). As regulatory authorities worldwide continue to develop and refine technical guidelines specific to EVs-based therapeutics, international organizations such as the International Council for Harmonization of Technical Requirements for Pharmaceuticals for Human Use (ICH) are expected to play a pivotal role in fostering regulatory convergence and promoting the global harmonization of standards for EVs-based drug products.
